# Improved workflows for high throughput library preparation using the transposome-based nextera system

**DOI:** 10.1186/1472-6750-13-104

**Published:** 2013-11-20

**Authors:** Sarah Lamble, Elizabeth Batty, Moustafa Attar, David Buck, Rory Bowden, Gerton Lunter, Derrick Crook, Bassam El-Fahmawi, Paolo Piazza

**Affiliations:** 1 Wellcome Trust Centre for Human Genetics, OX3 7BN Oxford, UK; 2 Nuffield Department of Medicine, University of Oxford, John Radcliffe Hospital, Headley Way, OX3 9DU Oxford, UK; 3 Oxford NIHR Biomedical Research Centre, John Radcliffe Hospital, Headley Way, OX3 9DU Oxford, UK; 4 Axygen Inc., A corning Subsidiary, 33120 Central Avenue, 94587 Union City, CA, USA

**Keywords:** Nextera, High-throughput, Library preparation, Sequencing, Normalisation

## Abstract

**Background:**

The Nextera protocol, which utilises a transposome based approach to create libraries for Illumina sequencing, requires pure DNA template, an accurate assessment of input concentration and a column clean-up that limits its applicability for high-throughput sample preparation. We addressed the identified limitations to develop a robust workflow that supports both rapid and high-throughput projects also reducing reagent costs.

**Results:**

We show that an initial bead-based normalisation step can remove the need for quantification and improves sample purity. A 75% cost reduction was achieved with a low-volume modified protocol which was tested over genomes with different GC content to demonstrate its robustness. Finally we developed a custom set of index tags and primers which increase the number of samples that can simultaneously be sequenced on a single lane of an Illumina instrument.

**Conclusions:**

We addressed the bottlenecks of Nextera library construction to produce a modified protocol which harnesses the full power of the Nextera kit and allows the reproducible construction of libraries on a high-throughput scale reducing the associated cost of the kit.

## Background

In the race for the first $1,000 human genome, next-generation, high-throughput sequencers such as the Illumina HiSeq instrument have been developed that can produce tens of gigabases of raw sequence per day. Such high outputs are essential for human whole-genome sequencing but excessive for many other applications, where the same amount of data would be sufficient to sequence several samples in applications such as targeted sequencing, microbial genome sequencing, RNAseq, ChIP-seq and amplicons. On existing short-read sequencing platforms, the main bottleneck in processing large numbers of samples is preparing them for loading: there is an acute need for low-cost, high-throughput, highly-multiplexed library production methods that moreover require only small amounts of input material.

Typically, library construction involves random fragmentation of starting DNA followed by the ligation of adapter oligos to support the amplification and sequencing of each molecule. Recently, Epicenter (now a subsidiary of Illumina) introduced Nextera, a library construction method [1] that combines simultaneous fragmentation of DNA and ligation of adapter sequences in a single reaction mediated by a transposase loaded with adapter oligos [1]. This technique, referred to as tagmentation, can produce high-quality genomic or cDNA libraries from as little as 20 pg DNA [2], reducing both preparation time and input material [2-6]. However, the current Nextera protocol requires pure DNA template, an accurate assessment of input concentration and a column cleanup that together limit its applicability for high-throughput sample preparation.

Here we describe a workflow validated to be automation friendly, which relaxes the need for very clean and accurately measured DNA and which enables increased library preparation throughput. The starting point of our process is a bead-based normalisation of genomic DNA (gDNA), a step that replaces quantification by using a defined amount of DNA-binding beads to enforce a reproducible, input DNA quantity while also removing possible contaminants such as salts and proteins. A fixed volume of normalised sample is then used for library construction. To increase our laboratory’s throughput, we validated the use of the Nextera kit in reduced volumes compatible with a 384-well PCR plate. We also tested various alternatives to clean up columns. Finally, we developed a series of 96, 8-base index tags included in two sets of primers that allow the construction of Nextera libraries with a possible level of multiplexing of up to 9216 (96x96) samples. With the aid of a liquid handling robot, the method described here allows the production of 2x384 samples in a day at a cost comparable to or lower than alternative methods. Our protocol reduces the cost per sample 4-fold from standard Nextera, 3-fold from Illumina TruSeq and by almost half compared to the Nextera XT kit.

## Results and discussion

### gDNA sample normalisation

**Figure 1:**
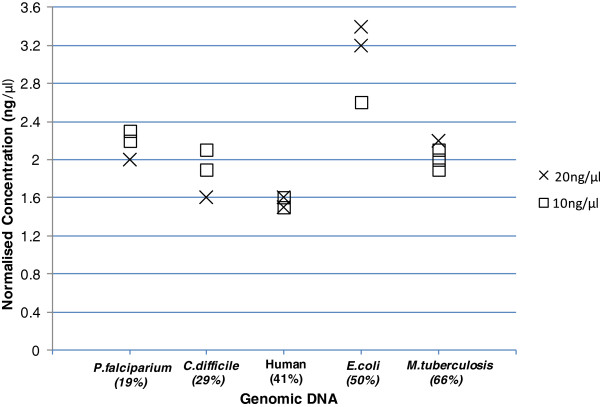
**Post-normalisation DNA concentrations.** DNA samples with input concentrations of approximately 20 ng/μl (×) and 10 ng/μl (□) were normalised with the Axygen PCR Normaliser kit. The output concentration is shown for each genome across a range of GC contents.

A major limitation of the Nextera protocol is the constraints it places on input samples. Accurate DNA quantification and high DNA quality are both important in achieving consistent tagmentation and reproducible library size distributions. Informally, we observed that samples with a turbid or otherwise abnormal appearance produced libraries with a shorter size distribution than intended, which were therefore unsuitable for sequencing. With both issues in mind, we evaluated the use of 3 kits prior to library preparation to remove inconsistencies between samples. The 3 kits were, AxyPrep Mag normalizer kit (Axygen Biosciences, Union City, CA, USA), DNA IQ System (Promega Corporation, Madison, WI 53711 USA) and Just-a-Plate PCR purification and normalisation kit (Charm Biotech, San Diego, CA 92130, USA). Initial trials proved the AxyPrep Mag kit to be the top performer; further evaluation was carried out on this kit alone. The kit, which is designed to normalise PCR products rather than genomic DNA (gDNA), was used with a modified protocol (Bassam El-Fahmawi, personal communication), on gDNA test samples from several organisms at two input concentrations (Figure 1). For human gDNA the normalised concentration ranged from 1.25-2.2 ng/μl (data not shown). The apparently modest normalisation performance was in fact comparable to that achieved using a more conventional method, Qubit (Invitrogen, Carlsbad, CA, USA). Similar results were attained across a range of genomes with GC contents (19 to 66%) indicating that the normalisation protocol was robust.

**Figure 2:**
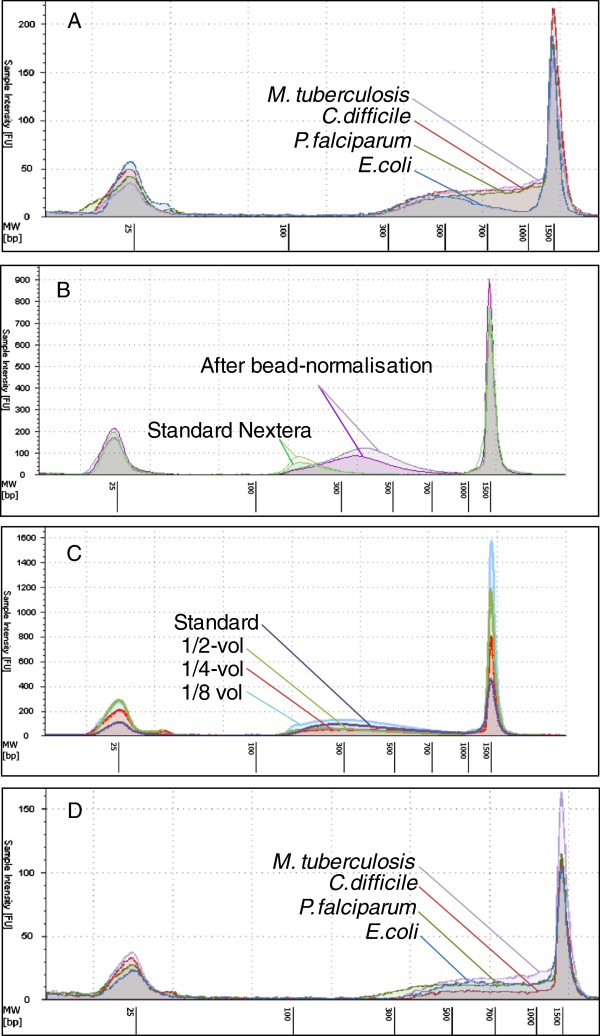
**Library QC Tape station electropherogram.** Nextera Post-PCR libraries constructed with a range of concentrations (post-normalisation) and gDNA from four different genomes: *Mycobacterium tuberculosis* (purple), *Escherichia coli* (blue), *Clostridium difficile* (red) and *Plasmodium falciparum* (green). Libraries were constructed using the standard Nextera protocol **(A)**. Evaluation of the Axyprep Mag Normaliser kit **(B)**: two individual *C. difficile* Nextera libraries were constructed using the standard Illumina protocol (light/dark green) and two with our normalisation workflow (light/dark purple). Where the standard library had very short inserts, our method produced a library with the normal size distribution. Evaluation of *C. difficile* Nextera Post-PCR libraries constructed using varying volume Nextera reactions **(C)**: standard (purple), half-volume (green), quarter-volume (red) and one-eighth volume (blue). Size distribution profiles of libraries constructed using normalisation followed by reaction E **(D)**: *M. tuberculosis* (purple), *E. coli* (blue), *C. difficile* (red) and *P. falciparum* (green).

Since none of the normalised samples exactly matched Illumina’s recommended 2.5 ng/μl for the Nextera kit, we evaluated the kit’s performance on a range of concentrations spanning from 1.5 to 3.4 ng/μl. Although the performance of the Nextera protocol is reported to depend strongly on input DNA concentration [6,7], little variation in the final size distribution of the library was observed (Figure 2A). To test whether the bead normalisation also removes impurities detrimental to Nextera library construction we took two samples of *Clostridium difficile* (*C. difficile*) gDNA which had a turbid appearance and prepared two standard Nextera libraries for each where one followed the bead-normalisation. Where the standard libraries had very small inserts, following gDNA normalisation we obtained libraries with the normal size distribution (Figure 2B). The most parsimonious explanation is that other factors such as contaminants present in the sample could have a greater effect than the absolute amount of DNA used in the reaction. Conceivably, the small insert sizes of the standard library suggests that only a small proportion of DNA was accessible to the transposase, altering the ideal ratio of DNA to enzyme. While the exact mechanism for this effect is unknown, these results support the idea that DNA purity is important, and more importantly, provide a practical way of improving the robustness of library construction when sample quality is variable, while completely removing the need for sample quantification.

### Low-volume nextera library construction over a range of GC contents

Performing bead-normalisation before Nextera library construction allows improvements in sample handling efficiency. To further increase throughput we sought to scale down the reaction volumes, making library construction possible even in 384-well PCR plates. We conducted a pilot study with gDNA from *C. difficile*; a species we sequence at large scales and for which we had an internal need for an improved workflow. As a follow up experiment, other organisms of clinical relevance were also tested. We evaluated the robustness of the Nextera kit in producing high-quality sequencing libraries using reduced volume reactions. Initial tests were performed with using full (A), half (B), quarter (C) and one-eighth (D) scaled tagmentation reactions with proportionately reduced input DNA amounts (Table 1). Since the kit comes with transposase pre-loaded with adapters and a proprietary buffer, reducing all reaction components in the same proportions as the input DNA also removes the need for custom preparation of buffers. The tagmentation reactions were cleaned up using Zymo Clean & Concentrate™ columns in which the elution volume was related to the initial reaction volume (see Methods), except for reaction D for which the recommended minimum column elution volume (6 μl) was used. Because elution volumes were also used to scale the subsequent PCR reactions, the effect of PCR volume on yield was checked by comparing the one-eighth-scale tagmentation reaction with a duplicate amplified in a full (50 μl) volume PCR (Reaction E). Following amplification and PCR clean-up, the quantities and size distributions of the libraries were compared. All test volumes produced libraries with a similarly broad peak ranging from 150-900 bp (Figure 2C), implying that a one-eighth volume Nextera tagmentation reaction containing only 6.25 ng of DNA could produce a library with characteristics similar to a standard 50 ng Nextera reaction. The one-eighth volume was evaluated across all genomes (Figure 2D) and produced a broad peak similar to the standard reaction (as seen in Figure 2A).

**Table 1:** Modified Nextera reaction volumes

	** Volumes (μl)**			
	**Tagmentation**	**Elution**	**PCR**	**Elution (2)**
Standard/Reaction A	50	25	50	32.5
Reaction B	25	12.5	25	16
Reaction C	12.5	6.5	12.5	10
Reaction D	6.25	6.5	12.5	10
Reaction E	6.25	25	50	10

Reduced volume Nextera reactions were evaluated. From left to right, the different columns show final volumes for the Tagmentation reaction, elution after purification, PCR amplification and elution after PCR purification respectively. DNA input amounts were scaled from the Illumina recommended amounts proportionately to the final volume of Tagmentation: full (Reaction A), half (Reaction B), quarter (Reaction C) and one-eighth (Reaction D and E). For Reaction E only the tagmentation reaction is reduced, followed by a standard PCR to increase yield.

Analysis of sequencing data revealed no functionally significant biases introduced by the use of reduced volumes in Nextera library preparation. Our finding is in agreement with previously published work which has shown that it is possible to produce libraries of acceptable complexity with 1-10 ng of gDNA [5] and that libraries can be made with even as little as 10 pg of DNA, albeit with decreased complexity [2,4-6]. Such low input requirements benefit studies of difficult to culture organisms (e.g. *M. tuberculosis* or non-model species) or limiting starting material (e.g. biopsies). Moreover, even in cases where DNA can be obtained in large amounts, a low input requirement allows other types of analysis to be performed on the same sample such as validation or follow up studies. We found that reduced volume reactions can also be applied to the Nextera XT kit allowing for even lower gDNA input when the sample is particularly limiting (data not shown).

Although the tagmentation reaction can be reduced 8-fold, we found that the standard PCR amplification of a quarter-scale reaction produced more concentrated libraries (Additional file 1: Figure S1), rendering QC steps easier to perform and interpret and avoiding the risks of duplication and AT-bias attached to increasing the number of PCR cycles. Nevertheless, the difference in volumes used for tagmentation and PCR reactions meant that we had to design a set of custom PCR primers, allowing us to maximise the use of the kit (Table 1).

**Figure 3:**
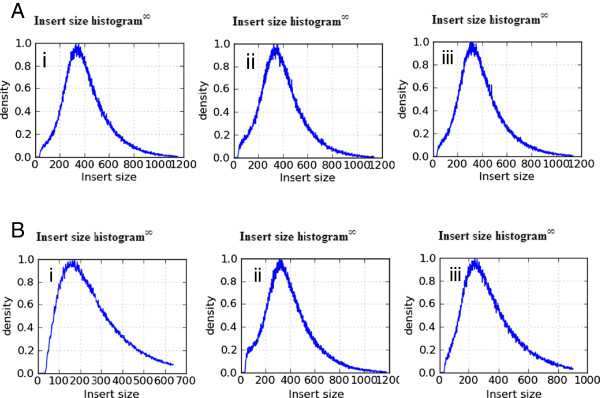
**Library insert size.** Libraries were constructed using the standard **(A)** or reaction E Nextera **(B)**. Sequencing metrics showed the library insert sizes for *C. difficile* (i), *E. coli* (ii) and *M. tuberculosis* (iii) to be approximately 250-300 bp irrespective of the original TapeStation profile (Figure 2A and B). *C. difficile* produced a library with a shorter size distribution (~180 bp) in this experiment (Bi).

GC content has been reported to influence Nextera kit efficiency [1]. To find out whether this effect was reproduced with our modified protocol (Additional file 2: Figure S2) and to identify the range of organisms for which our protocol would be useful we sequenced four organisms with a range of GC contents. DNA samples from *Plasmodium falciparum* (19%), *Clostridium difficile* (29%)*, Escherichia* coli (50%)*,* and *Mycobacterium tuberculosis* (66%) were normalised to a mean 2.1 ng/μl final sample concentration (Figure 1). *E. coli* (50%GC) produced noticeably higher output concentrations (2.6-3.4 ng/μl). 2.5 μl of normalised DNA (approximately 5.5 ng, except for the *E. coli* reactions with 7.6 ng DNA) was used in the one-eighth-scale (6.2 μl) tagmentation reaction. For each organism, reduced-volume libraries were produced in triplicate and compared with the standard 50 ng Nextera prep. Analysis of library size distributions prior to sequencing implied that transposase efficiency was comparable across the range of DNA concentrations GC content used in this study (Figure 2A, C and D). One Nextera standard and one low volume library from each genome were pooled and sequenced on a Miseq 150b PE run. Sequencing metrics revealed library insert sizes of 250-300 bp (Figure 3) for *C. difficile*, *E. coli* and *M. Tuberculosis*, irrespective of the original TapeStation profile (Figure 2A and 2D). The data obtained in this experiment showed a shift in the size distribution between the standard and modified workflow of the *C. difficile* library (3Ai and Bi). In particular, the modified workflow produced a library with an insert size below 200 bp, however, subsequent libraries prepared in the same way for *C. difficile* showed insert sizes of 250-300 bp (data not shown) indicating an intrinsic variability in library sizes obtained by tagmentation. The *P.falciparum* library was extremely biased and produced unusable data.

**Figure 4:**
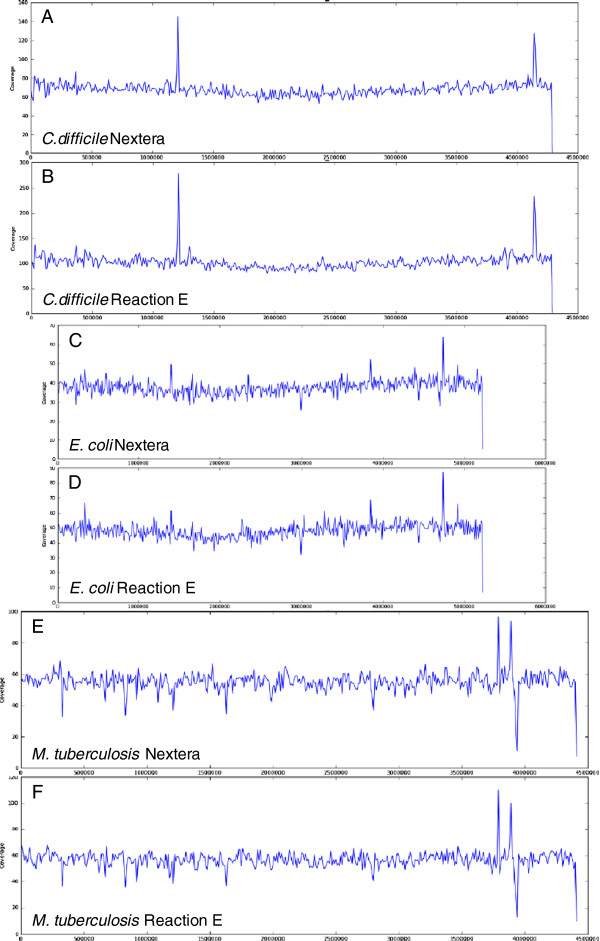
**Sequencing QC: genome coverage.** Coverage across the genome for *C. difficile***(A,B)**, *E. coli***(C,D)** and *M. tuberculosis***(E,F)** libraries constructed with standard **(A,C,E)** and reaction E **(B,D,F)** Nextera preparation. The coverage is consistent between the two preparations, with both protocols producing good, even coverage across the genome. The large spikes seen in all three genomes probably represent repetitive regions and are consistent between the two preparations.

We used mapping- and *de novo* assembly-based analysis of sequencing data to compare the low-volume Nextera prep and the standard prep for each of the four genomes (metrics in Table 2). For *M. tuberculosis*, *E. coli* and *C. difficile*, no functionally significant differences were evident between the standard and low-volume Nextera preps. We observed high and comparable genome coverage in both low-volume and standard preps (Figure 4). No single-nucleotide differences were identified between prep types using our standard mapping-based basecalls filtered as in [8]. There was a small GC-bias in coverage (Table 2) although its magnitude was small considering the wide range of GC content in the study (Additional file 3: Figure S3). Both the standard Nextera and our modified protocol showed a similar under-representation at very high GC (>80%) and failed to produce acceptable libraries at very low GC (<20%). PCR amplification during Illumina library preparation has been previously shown to cause GC bias after sequencing, emphasizing the value of PCR-free library methods for such organisms [9-11]. The transposase is known to have a particular insertion preference which was reported to introduce a low level bias [1]. Our data showed a pattern at the beginning (first 10 bases) of each read which confirms that the transposase has a preference for insert sites within AT rich regions [12]; however, we were unable to detect any major consequences of this in any further downstream analysis. Our preliminary attempts to improve representation at the ends of the GC spectrum by substituting the Nextera PCR Master Mix with either of two enzymes, Q5 (New England Biolabs) or HiFi (Kapa) were unsuccessful despite the fact that both enzymes have been reported to produce more even representation of the genome under standard conditions or with the addition of TMAC [9,10]. Due to the formulation of the Nextera kit it was impossible to test a PCR free approach, however, our results with two alternative PCR enzyme support the hypothesis that at least a component of the GC bias observed in *P.falciparum* is due to the transposase insertion mechanism.

**Table 2:** Data metrics from MiSeq sequencing of standard and modified Nextera library preparations

	**Genomes trial**							
**Reference genome**	***P. falciparum***		***C. difficile***		***E. coli***		***M. tuberculosis***	
**% GC**	***19***		***29***		***50***		***66***	
**Size (Mb)**	***23.3***		***4.3***		***5.2***		***4.4***	
**Sample**	Standard	Reaction E	Standard	Reaction E	Standard	Reaction E	Standard	Reaction E
**Yield (Mb) Q20**	432	260	278	457	171	220	184	194
**% Mapped Reads**	96.6	96.8	97.8	98.4	96.5	96.6	95.6	96.0
**% Duplicates**	0.5	0.5	0.8	1.6	0.4	0.7	0.5	0.6
**% GC**	22.7	22	28.6	30.4	48.4	48.6	61.8	61.8

Genome trial data metrics. Samples were pooled and sequenced on a 150 bp PE Miseq Run. Data from the standard and the modified reaction were compared and showed no significant differences between the library preparations.

### High-throughput low volume library construction

To fully harness the power of the Nextera kit the whole protocol needs to be capable of automation. The standard clean-up method using columns to remove tagmentation reaction constituents hinders the use of robotics, although throughput can be increased with the use of a 96 well manifold (Zymo). We compared alternative clean-up procedures in order to find a comparably-performing replacement for the column step. The three methods (1) column purification (Zymo), (2) AMPure XP, and (3) QG/AMPure XP were performed in duplicate. All clean-up systems produced libraries of a similar quantity and size profile, indicating that a bead-based clean-up method can be a suitable replacement for columns (Additional file 4: Figure S4). When the clean-up step is removed entirely and the tagmented DNA put straight into the PCR reaction, a library with a good profile is still produced (Additional file 4: Figure S4). Others have promoted the routine use of AMPure XP in library preps [6] and a clean-up before PCR may not even be necessary [1]. Since the transposase is not able to “disengage” in vitro [13] we hypothesise that heat inactivation of the enzyme occurs during the early steps of PCR. In summary, the replacement of the column clean-up with a bead-based approach provided the final element that allowed full automation of the Nextera workflow using our current instruments. Interestingly, when testing different methods to clean the tagmentation reaction, we found that even a simple Ampure XP bead clean-up or immediate transfer into the PCR mix were successful reinforcing the idea that inactivation of the transposase can occur during PCR.

### In house 96 tag primers

In our lab we routinely use a set of 96 custom indices (Additional file 5: Figure S5) for conventional paired end sequencing. We aimed to design a set of indices that would allow single sequencing errors to be corrected, and double errors to be detected. In addition, we required (i) nucleotide use to be balanced across indices, to avoid low complexity issues when using subsets of indices and prevent increased error rates, and (ii) no nucleotide triplets to occur anywhere, and no duplets to occur at either end, to avoid potential increased error rates within homopolymers. Using the quaternary Hamming code (as described in Methods) we obtained a list of 120 indices. We picked 96 of these to form our actual index set. Every two indices differ in at least 4 positions, allowing single errors to be corrected, and double errors to be detected but not necessarily corrected. Triple errors can in principle be mistaken for single errors and be mis-corrected in this way; however, only 1.5% (936/60480) of possible triple errors would in fact be mis-corrected. By not using reads whose indices include likely erroneous bases, the misclassification rate will be negligible. The readability of the tags has been tested and they are routinely used. By combining the new Nextera oligo design with dual indexing and our tags we can expand our current multiplexing capacity to up to 96x96 samples in a pool.

## Conclusions

Transposome-based preparation of genomic libraries for high-throughput sequencing (Nextera) provides a convenient and quick alternative to conventional methods that, moreover, needs only relatively little input material. However, in its currently available form, Nextera is not robust to variation in sample concentration and quality, is not easily automatable, and is substantially more expensive than conventional preps. In this work, we describe the validation of modifications to the standard Nextera protocol which solve these problems and make it possible to adopt fast Nextera protocols as the standard for large-scale microbial genome sequencing and similar applications. We made a marked improvement to the protocol by providing a series of strategies that address all the identified bottlenecks.

Firstly we applied a bead-based normalisation step to decrease sample concentration variability which leads to an increase in library quality and removes the need for quantification. We validated the normalisation protocol over genomes with different GC content or complexity. Secondly, in order to reduce costs and input DNA amount, we lowered the volume of the Nextera library preparation. Sequencing data analysis of the modified protocol revealed no functionally significant biases to the majority of the data and good coverage across the whole genome was achieved. On the other hand, our results illustrated that the Nextera kit is not ideal for low GC genomes due to the compulsory PCR step and the transposase preference for AT rich sequences.

The normalisation prior to Nextera library construction is not an absolute requirement; if the DNA is scarce and of good quality it can be used directly with the reduced volume protocol to keep the sample input to a minimum. In addition to these two modifications we also replaced the column clean-up with an automatable bead-based approach which allows increased throughput. Finally we designed custom primers and multiplex tags to increase throughput to 96x96 samples.

We addressed the bottlenecks of Nextera library construction to produce a modified protocol which harnesses the full power of the Nextera kit and allows the reproducible construction of libraries on a high-throughput scale reducing the associated cost of the kit.

## Methods

### Pre-library normalisation

gDNA was normalised using the AxyPrep Mag PCR Normalizer Kit (Axygen Biosciences). 60 μl of AxyPrep Mag normalizer was added to 20 μl of gDNA, pipette mixed and then left gently shaking for 5 minutes. The samples were placed on a magnet for 2 minutes before the supernatant was removed. Whilst still on the magnet the beads were washed with 100 μl of distilled water without resuspending the beads. The water was then discarded. The samples were removed from the magnet and eluted in 25 μl of freshly prepared 10 mM NaOH by fully resuspending the beads and shaking gently for 5 minutes. After placing the samples on the magnet for 2 minutes the supernatant was removed and neutralised with 10 μl of 20 mM Tris pH 7. The concentration of the normalized samples was determined by Qubit (Invitrogen) following the manufacturers specifications.

### Reduced volume nextera library preparation

Nextera libraries were constructed using *Clostridium difficile* gDNA and the Illumina Nextera™ Kit. A standard tagmentation reaction (A) was set up to a final volume of 50 μl according to the Nextera protocol. Additional reactions were performed where the final volume and all the reagents, including input DNA, were proportionally reduced: (B) 25 μl, (C) 12.5 μl, (D) 6.25 μl and (E) 6.25 μl (Table 1). All reactions were set up in duplicate and incubated as per the manufacturer’s instructions. Reactions were cleaned up using DNA Clean & Concentrate™ (Zymo Research) according to the manufacturer’s instructions. Elution volumes were as in Table 1. Standard PCR reactions were setup according to the Nextera protocol. All reagents in the reduced volume PCR reactions were decreased proportionally except reaction D (Table 1). Thermocycling was carried out on a Tetrad (Bio-Rad, 1000 Alfred Nobel Drive, Hercules, CA, 94547, USA) with the following standard Nextera parameters: PCR clean-up was performed following the Nextera protocol using a 0.6:1 ratio of AMPure XP® (Beckman Coulter) to PCR reaction. Reactions were eluted with EB (Qiagen).

### Library quantification and size determination

Nextera libraries were quantified using Qubit and the size profile was analysed on the 2200 TapeStation (Agilent). For libraries with concentrations below 3 ng/μl the High Sensitivity (HS) ScreenTape was used. Final pooled libraries were quantified by qPCR using Brilliant III SYBR Green qPCR Master Mix (Agilent).

### Nextera library prep using four genomes

gDNA was quantified using Qubit (Invitrogen). Using the recommended 50 ng gDNA a standard Nextera library was constructed for each genome. Additionally, five aliquots of each genome were normalised using AxyPrep Mag PCR Normalizer Kit, see above. The normalised samples were quantified using Qubit and three from each genome were selected for library construction. Nextera library prep was performed using the 6.25 μl reaction described above (reaction E). 2.5 μl of bead-normalised DNA was used in the prep.

### Pooling and sequencing

The libraries that were selected for sequencing were normalised using qPCR or Qubit readings and pooled together accordingly. The pooled library was diluted to ~10nM for storage and quantification via real-time PCR. The 10nM library was denatured and further diluted prior to loading on a MiSeq paired-end 150-bp (v1) sequence run.

### Tagmentation reaction clean-up

Tagmentation reactions were set up using the reaction E protocol (above) with *C. difficile* gDNA at 2.5 ng/μl. Clean-up was performed in duplicate using four different methods: (1) Zymo column purification according to the manufacturer’s instructions, (2) 11 μl AMPure XP according to manufacturer’s instructions, (3) the addition of 9 μl of QG buffer (Qiagen) before clean-up with 27 μl AMPure XP and (4) no clean-up, the reaction was put straight onto ice and the tagmented DNA was then used directly in a 12.5 μl Nextera PCR (reaction D). 1–3 were eluted in 25 μl of EB (reaction E). PCR was carried out using 25 μl KAPA HiFi 2X master mix (KAPA), 2 μl custom Primers(10 μM), 20 μl tagmented DNA and nuclease-free water up to 50 μl. Reactions were thermocycled on a Tetrad following KAPA’s recommended Nextera protocol; clean-up was performed as standard. Libraries were compared using the 2200 TapeStation.

### PCR with alternative polymerases

gDNA samples were normalised using AxyPrep and libraries were constructed in duplicate following Nextera library prep reaction D. PCR was performed under standard Nextera conditions for the control samples. Additional reactions were performed in duplicate with two enzymes not supplied in the Nextera kit. The reactions were as follows: 6.25 μl KAPA HiFi 2X Master Mix (KAPA) or NEBNext High-Fidelity 2X PCR Master Mix (NEB), 1.25 μl custom primers, 5 μl tagmented DNA. Reactions were thermocycled on a Tetrad following the recommended protocol.

### Data analysis

Reference genomes were obtained from GenBank for *E. coli* strain CFT073 (accession NC_004431), *M. tuberculosis* strain H37Rv (accession NC_000962), and *C. difficile* strain CD630 (AM180355) and from the Wellcome Trust Sanger Institute for *P. falciparum* 3D7 (ftp://ftp.sanger.ac.uk/pub/pathogens/Plasmodium/falciparum/3D7/3D7.version2.1.5/Pf3D7_v2.1.5.fasta). Reads were mapped to the reference genomes using Stampy [14] v1.0.18 without BWA pre-mapping and with a substitution rate off 0.01. Single nucleotide variants were called as previously described in Eyre et al. [15]. Briefly, variants were called using the samtools v1.0.12-10. [16] mpileup command with options “-M0 -Q30 -q30 -o40 -e20 -h100 -m2 -D –S” and filtered to remove variants which were not well-supported or fell in repetitive regions. Genomes were assembled using Velvet v1.0.11. [17] VelvetOptimiser was used to determine hash size and coverage parameters to maximize n50 for the assembly (Zerbino 2010). The quality of the genome assemblies was assessed using Mauve Assembly Metrics [18]. Genome coverage data was determined using the Genome Analysis Toolkit [19]. Sequencing data quality was assessed using FastQC (http://www.bioinformatics.babraham.ac.uk/projects/fastqc/). GC bias plots were produced using Picard (http://picard.sourceforge.net/). All other analysis was performed using custom Python scripts. All data can be found at http://www.ebi.ac.uk/ena/data/view/PRJEB4315.

### In house 96 tag primer design

To design a set of indices to meet our requirements, we used the quaternary Hamming (8,4) code, with length 8 and 4 parity characters, giving 4^4 = 256 code words each consisting of 8 characters from the alphabet [1]. Regarded as 8-nucleotide DNA words, this code contains many length-3 homopolymers. To address this issue, we first chose an arbitrary length-8 word W, and added character W-_i_ (modulo 4) to each i-th character of the code, and converted this into the DNA alphabet. From the resulting indices we removed those containing 2 or 3 identical consecutive nucleotides at either end. Finally, we varied W to select index sets that showed balanced nucleotide use in each position, and maximized the number of indices. Using W = (1,2,2,3,3,0,0,0), we obtain a list of 120 indices satisfying all criteria, 96 of which form our in-house index set.

## Competing interests

The author(s) declare that they have no competing interests.

## Authors’ contributions

Research in the lab was carried out by SL, PP, and MA. Bioinformatic analyses were carried out by LB. Custom indices were designed by GL. The project was conceived by DB, RB, PP, and DC. The normalisation protocol was provided by BEF. The manuscript was written by SL, PP, RB and DB. All authors read and approved the final manuscript.

## Supplementary Material

Additional file 1: Figure S1 Increased PCR volume QC. Nextera libraries constructed with one-eighth volume tagmentation reaction were subjected to different PCR volumes: standard PCR-Reaction D (green) and one-fourth volume PCR-Reaction E (purple). Click here for file

Additional file 2: Figure S2 Modified Nextera workflow improvements. Schematic representation of the Nextera workflow with a summary of the improvements obtained for each step. Click here for file

Additional file 3: Figure S3 GC Bias QC. GC bias metrics from Picard for (A) *C. difficile* libraries, (B) *E. coli* libraries, (C) *M. tuberculosis* libraries prepared using the standard (1) and reaction E (2) Nextera prep. Blue dots show coverage against different GC windows. Click here for file

Additional file 4: Figure S4 Library QC following different tagmentation reaction cleanup techniques. Libraries were constructed and the tagmentation clean-up was performed using zymo columns (blue), Ampure XP (red), Ampure XP with QG buffer (green) or no clean-up (purple). All methods produced similar profiles with a slight shift observed when the clean-up was eliminated. Click here for file

Additional file 5: Figure S5 Index primers. A list of the primers and indices validated is provided. Click here for file
