# Supplementary figures and images for: Improved workflows for high throughput library preparation using the transposome-based nextera system
Source: BMC Biotechnol. 2013 Nov 20;13:104. doi: 10.1186/1472-6750-13-104 (PMC4222894; doi:10.1186/1472-6750-13-104)

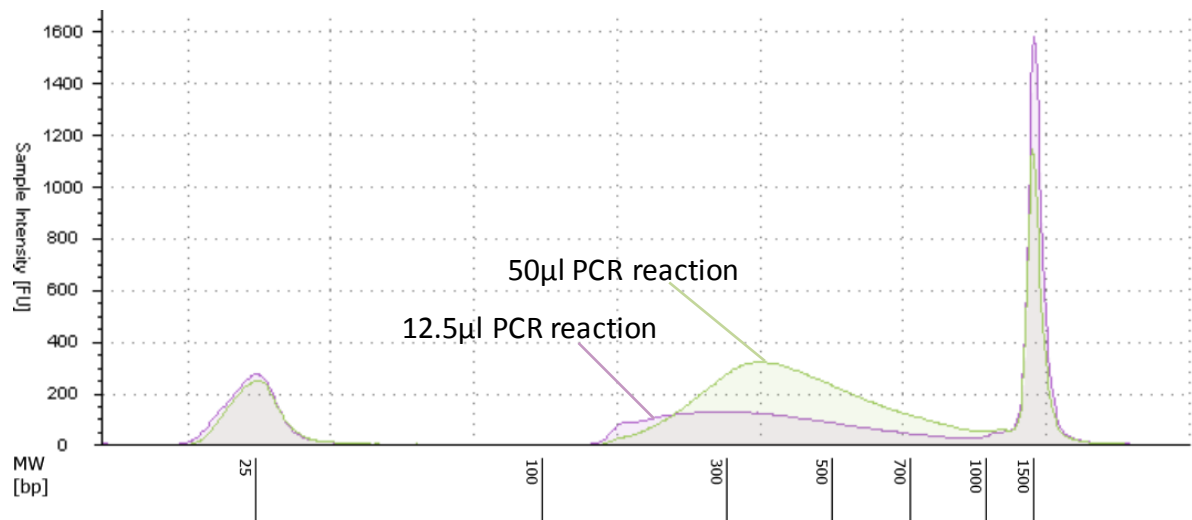

Supplement: Additional file 1: Figure S1 — Increased PCR volume QC. Nextera libraries constructed with one-eighth volume tagmentation reaction were subjected to different PCR volumes: standard PCR-Reaction D (green) and one-fourth volume PCR-Reaction E (purple). [file 1472-6750-13-104-S1.pdf]

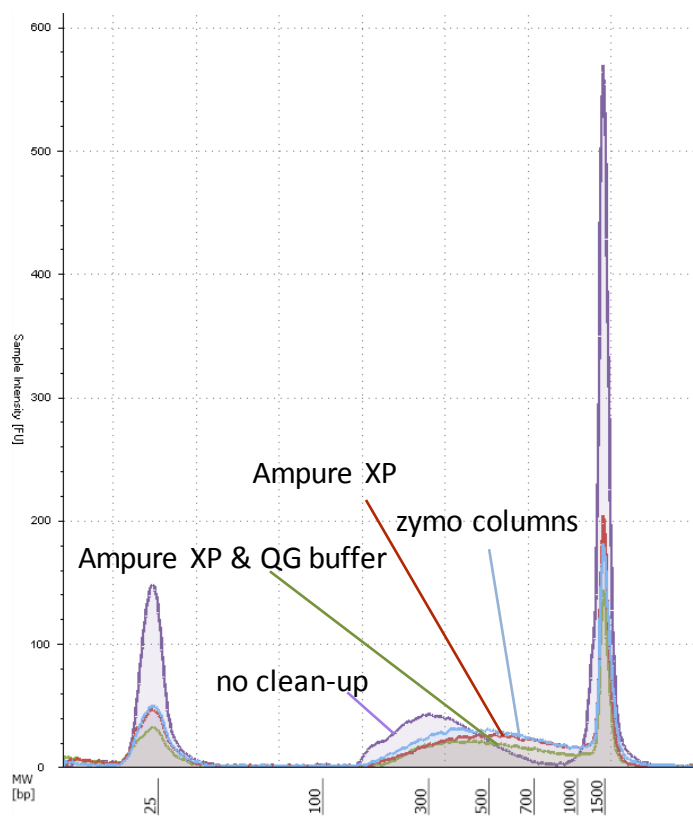

Supplement: Additional file 4: Figure S4 — Library QC following different tagmentation reaction cleanup techniques. Libraries were constructed and the tagmentation clean-up was performed using zymo columns (blue), Ampure XP (red), Ampure XP with QG buffer (green) or no clean-up (purple). All methods produced similar profiles with a slight shift observed when the clean-up was eliminated. [file 1472-6750-13-104-S4.pdf]
