# Supplementary material for: Improved workflows for high throughput library preparation using the transposome-based nextera system
Source: BMC Biotechnol. 2013 Nov 20;13:104. doi: 10.1186/1472-6750-13-104 (PMC4222894; doi:10.1186/1472-6750-13-104)
Supplement: Additional file 2: Figure S2 — Modified Nextera workflow improvements. Schematic representation of the Nextera workflow with a summary of the improvements obtained for each step. [file 1472-6750-13-104-S2.pdf]

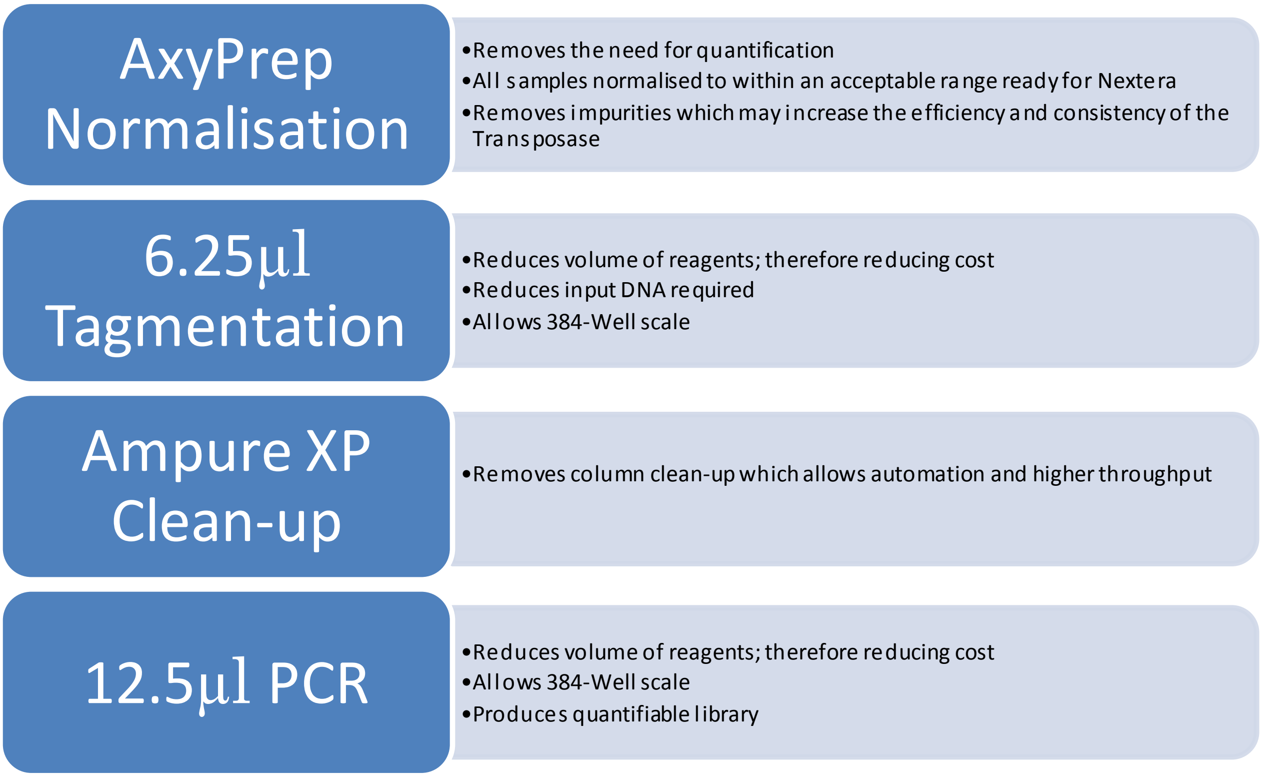

Supplementary Figure 2 Modified Nextera workflow improvements.
