# Supplementary material for: Improved workflows for high throughput library preparation using the transposome-based nextera system
Source: BMC Biotechnol. 2013 Nov 20;13:104. doi: 10.1186/1472-6750-13-104 (PMC4222894; doi:10.1186/1472-6750-13-104)
Supplement: Additional file 3: Figure S3 — GC Bias QC. GC bias metrics from Picard for (A) C. difficile libraries, (B) E. coli libraries, (C) M. tuberculosis libraries prepared using the standard (1) and reaction E (2) Nextera prep. Blue dots show coverage against different GC windows. [file 1472-6750-13-104-S3.pdf]

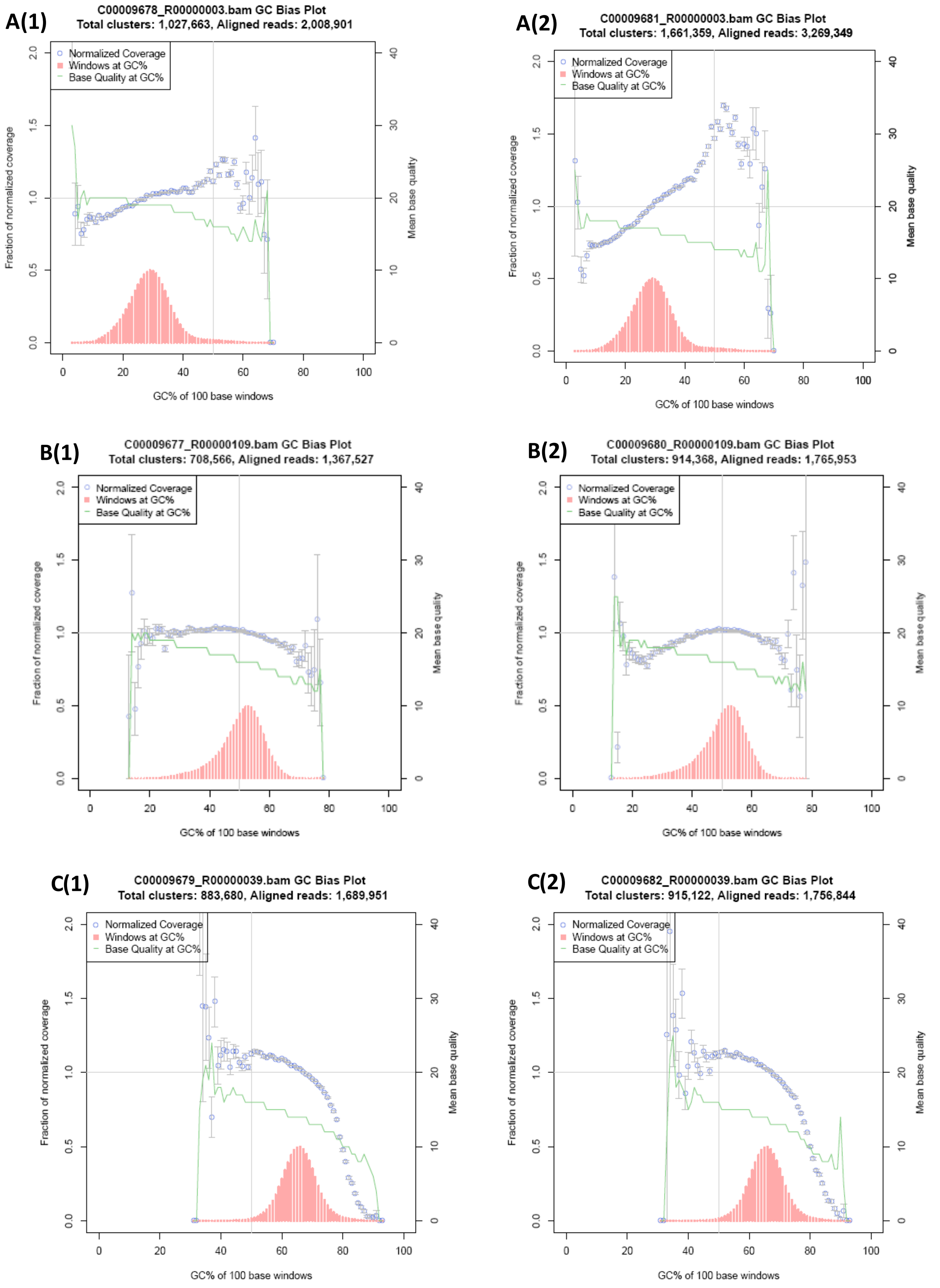

A(1)
C00009678_R00000003.bam GC Bias Plot
Total clusters: 1,027,663, Aligned reads: 2,008,901
A(2)
C00009681_R00000003.bam GC Bias Plot
Total clusters: 1,661,359, Aligned reads: 3,269,349
B(1)
C00009677_R00000109.bam GC Bias Plot
Total clusters: 708,566, Aligned reads: 1,367,527
B(2)
C00009680_R00000109.bam GC Bias Plot
Total clusters: 914,368, Aligned reads: 1,765,953
C(1)
C00009679_R00000039.bam GC Bias Plot
Total clusters: 883,680, Aligned reads: 1,689,951
C(2)
C00009682_R00000039.bam GC Bias Plot
Total clusters: 915,122, Aligned reads: 1,756,844
Normalized Coverage
Windows at GC%
Base Quality at GC%
Fraction of normalized coverage
Mean base quality
GC% of 100 base windows
