# Supplementary material for: Improved workflows for high throughput library preparation using the transposome-based nextera system
Source: BMC Biotechnol. 2013 Nov 20;13:104. doi: 10.1186/1472-6750-13-104 (PMC4222894; doi:10.1186/1472-6750-13-104)
Supplement: Additional file 5: Figure S5 — Index primers. A list of the primers and indices validated is provided. [file 1472-6750-13-104-S5.pdf]

	Index	Index Primer Sequence
1	AGTTAACA	CAA GCA GAA GAC GGC ATA CGA GAT AGT TAA CAG TGA CTG GAG TTC AGA CGT GTG CTC TTC CGA TC*T
2	ACGCGCGA	CAA GCA GAA GAC GGC ATA CGA GAT ACG CGC GAG TGA CTG GAG TTC AGA CGT GTG CTC TTC CGA TC*T
3	CTCTTCTA	CAA GCA GAA GAC GGC ATA CGA GAT CTC TTC TAG TGA CTG GAG TTC AGA CGT GTG CTC TTC CGA TC*T
4	GAGAGAGC	CAA GCA GAA GAC GGC ATA CGA GAT GAG AGA GCG TGA CTG GAG TTC AGA CGT GTG CTC TTC CGA TC*T
5	CAGTACTC	CAA GCA GAA GAC GGC ATA CGA GAT CAG TAC TCG TGA CTG GAG TTC AGA CGT GTG CTC TTC CGA TC*T
6	CAATCAAG	CAA GCA GAA GAC GGC ATA CGA GAT CAA TCA AGG TGA CTG GAG TTC AGA CGT GTG CTC TTC CGA TC*T
7	GTATATAG	CAA GCA GAA GAC GGC ATA CGA GAT GTA TAT AGG TGA CTG GAG TTC AGA CGT GTG CTC TTC CGA TC*T
8	GATTAATG	CAA GCA GAA GAC GGC ATA CGA GAT GAT TAA TGG TGA CTG GAG TTC AGA CGT GTG CTC TTC CGA TC*T
9	GACCAACA	CAA GCA GAA GAC GGC ATA CGA GAT GAC CAA CAG TGA CTG GAG TTC AGA CGT GTG CTC TTC CGA TC*T
10	TGTGGCGA	CAA GCA GAA GAC GGC ATA CGA GAT TGT GGC GAG TGA CTG GAG TTC AGA CGT GTG CTC TTC CGA TC*T
11	TCTCTCTA	CAA GCA GAA GAC GGC ATA CGA GAT TCT CTC TAG TGA CTG GAG TTC AGA CGT GTG CTC TTC CGA TC*T
12	AGAGGAGC	CAA GCA GAA GAC GGC ATA CGA GAT AGA GGA GCG TGA CTG GAG TTC AGA CGT GTG CTC TTC CGA TC*T
13	ACTAACTC	CAA GCA GAA GAC GGC ATA CGA GAT ACT AAC TCG TGA CTG GAG TTC AGA CGT GTG CTC TTC CGA TC*T
14	ACCACAAG	CAA GCA GAA GAC GGC ATA CGA GAT ACC ACA AGG TGA CTG GAG TTC AGA CGT GTG CTC TTC CGA TC*T
15	CACAATAG	CAA GCA GAA GAC GGC ATA CGA GAT CAC AAT AGG TGA CTG GAG TTC AGA CGT GTG CTC TTC CGA TC*T
16	AGCCAATG	CAA GCA GAA GAC GGC ATA CGA GAT AGC CAA TGG TGA CTG GAG TTC AGA CGT GTG CTC TTC CGA TC*T
17	GATTGGCA	CAA GCA GAA GAC GGC ATA CGA GAT GAT TGG CAG TGA CTG GAG TTC AGA CGT GTG CTC TTC CGA TC*T
18	CAATTGGA	CAA GCA GAA GAC GGC ATA CGA GAT CAA TTG GAG TGA CTG GAG TTC AGA CGT GTG CTC TTC CGA TC*T
19	TCGAAGTA	CAA GCA GAA GAC GGC ATA CGA GAT TCG AAG TAG TGA CTG GAG TTC AGA CGT GTG CTC TTC CGA TC*T
20	GACTTCGC	CAA GCA GAA GAC GGC ATA CGA GAT GAC TTC GCG TGA CTG GAG TTC AGA CGT GTG CTC TTC CGA TC*T
21	TGACACTC	CAA GCA GAA GAC GGC ATA CGA GAT TGA CAC TCG TGA CTG GAG TTC AGA CGT GTG CTC TTC CGA TC*T
22	TGGCCAAG	CAA GCA GAA GAC GGC ATA CGA GAT TGG CCA AGG TGA CTG GAG TTC AGA CGT GTG CTC TTC CGA TC*T
23	ACGCATAG	CAA GCA GAA GAC GGC ATA CGA GAT ACG CAT AGG TGA CTG GAG TTC AGA CGT GTG CTC TTC CGA TC*T
24	AGAACCTG	CAA GCA GAA GAC GGC ATA CGA GAT AGA ACC TGG TGA CTG GAG TTC AGA CGT GTG CTC TTC CGA TC*T
25	AGCCGGCA	CAA GCA GAA GAC GGC ATA CGA GAT AGC CGG CAG TGA CTG GAG TTC AGA CGT GTG CTC TTC CGA TC*T
26	ACCATGGA	CAA GCA GAA GAC GGC ATA CGA GAT ACC ATG GAG TGA CTG GAG TTC AGA CGT GTG CTC TTC CGA TC*T
27	CTAGAGTA	CAA GCA GAA GAC GGC ATA CGA GAT CTA GAG TAG TGA CTG GAG TTC AGA CGT GTG CTC TTC CGA TC*T
28	AGTCTCGC	CAA GCA GAA GAC GGC ATA CGA GAT AGT CTC GCG TGA CTG GAG TTC AGA CGT GTG CTC TTC CGA TC*T
29	GTCGACTC	CAA GCA GAA GAC GGC ATA CGA GAT GTC GAC TCG TGA CTG GAG TTC AGA CGT GTG CTC TTC CGA TC*T
30	GTTGCAAG	CAA GCA GAA GAC GGC ATA CGA GAT GTT GCA AGG TGA CTG GAG TTC AGA CGT GTG CTC TTC CGA TC*T
31	TGTGATAG	CAA GCA GAA GAC GGC ATA CGA GAT TGT GAT AGG TGA CTG GAG TTC AGA CGT GTG CTC TTC CGA TC*T
32	GAGGCCTG	CAA GCA GAA GAC GGC ATA CGA GAT GAG GCC TGG TGA CTG GAG TTC AGA CGT GTG CTC TTC CGA TC*T
33	AGAATTCA	CAA GCA GAA GAC GGC ATA CGA GAT AGA ATT CAG TGA CTG GAG TTC AGA CGT GTG CTC TTC CGA TC*T
34	TGGCTGGA	CAA GCA GAA GAC GGC ATA CGA GAT TGG CTG GAG TGA CTG GAG TTC AGA CGT GTG CTC TTC CGA TC*T
35	TCCTCTTA	CAA GCA GAA GAC GGC ATA CGA GAT TCC TCT TAG TGA CTG GAG TTC AGA CGT GTG CTC TTC CGA TC*T
36	AGGAAGGC	CAA GCA GAA GAC GGC ATA CGA GAT AGG AAG GCG TGA CTG GAG TTC AGA CGT GTG CTC TTC CGA TC*T
37	ACGTCGTC	CAA GCA GAA GAC GGC ATA CGA GAT ACG TCG TCG TGA CTG GAG TTC AGA CGT GTG CTC TTC CGA TC*T
38	ACATGCAG	CAA GCA GAA GAC GGC ATA CGA GAT ACA TGC AGG TGA CTG GAG TTC AGA CGT GTG CTC TTC CGA TC*T
39	TCGAGACG	CAA GCA GAA GAC GGC ATA CGA GAT TCG AGA CGG TGA CTG GAG TTC AGA CGT GTG CTC TTC CGA TC*T
40	AGTTGGTG	CAA GCA GAA GAC GGC ATA CGA GAT AGT TGG TGG TGA CTG GAG TTC AGA CGT GTG CTC TTC CGA TC*T
41	GAGGTTCA	CAA GCA GAA GAC GGC ATA CGA GAT GAG GTT CAG TGA CTG GAG TTC AGA CGT GTG CTC TTC CGA TC*T
42	GTTGTGGA	CAA GCA GAA GAC GGC ATA CGA GAT GTT GTG GAG TGA CTG GAG TTC AGA CGT GTG CTC TTC CGA TC*T
43	CTTCCTTA	CAA GCA GAA GAC GGC ATA CGA GAT CTT CCT TAG TGA CTG GAG TTC AGA CGT GTG CTC TTC CGA TC*T
44	GAAGAGGC	CAA GCA GAA GAC GGC ATA CGA GAT GAA GAG GCG TGA CTG GAG TTC AGA CGT GTG CTC TTC CGA TC*T
45	TGTACGTC	CAA GCA GAA GAC GGC ATA CGA GAT TGT ACG TCG TGA CTG GAG TTC AGA CGT GTG CTC TTC CGA TC*T
46	TGCAGCAG	CAA GCA GAA GAC GGC ATA CGA GAT TGC AGC AGG TGA CTG GAG TTC AGA CGT GTG CTC TTC CGA TC*T
47	CTAGGACG	CAA GCA GAA GAC GGC ATA CGA GAT CTA GGA CGG TGA CTG GAG TTC AGA CGT GTG CTC TTC CGA TC*T
48	GACCGGTG	CAA GCA GAA GAC GGC ATA CGA GAT GAC CGG TGG TGA CTG GAG TTC AGA CGT GTG CTC TTC CGA TC*T
49	TGATCAGA	CAA GCA GAA GAC GGC ATA CGA GAT TGA TCA GAG TGA CTG GAG TTC AGA CGT GTG CTC TTC CGA TC*T
50	ACATATGA	CAA GCA GAA GAC GGC ATA CGA GAT ACA TAT GAG TGA CTG GAG TTC AGA CGT GTG CTC TTC CGA TC*T
51	TCAACCAC	CAA GCA GAA GAC GGC ATA CGA GAT TCA ACC ACG TGA CTG GAG TTC AGA CGT GTG CTC TTC CGA TC*T
52	AGCTCTGC	CAA GCA GAA GAC GGC ATA CGA GAT AGC TCT GCG TGA CTG GAG TTC AGA CGT GTG CTC TTC CGA TC*T
53	GTACCGTC	CAA GCA GAA GAC GGC ATA CGA GAT GTA CCG TCG TGA CTG GAG TTC AGA CGT GTG CTC TTC CGA TC*T
54	GTGCGCAG	CAA GCA GAA GAC GGC ATA CGA GAT GTG CGC AGG TGA CTG GAG TTC AGA CGT GTG CTC TTC CGA TC*T
55	TCCTTCCG	CAA GCA GAA GAC GGC ATA CGA GAT TCC TTC CGG TGA CTG GAG TTC AGA CGT GTG CTC TTC CGA TC*T
56	AGGAGAAT	CAA GCA GAA GAC GGC ATA CGA GAT AGG AGA ATG TGA CTG GAG TTC AGA CGT GTG CTC TTC CGA TC*T
57	GTCACAGA	CAA GCA GAA GAC GGC ATA CGA GAT GTC ACA GAG TGA CTG GAG TTC AGA CGT GTG CTC TTC CGA TC*T
58	TGCAATGA	CAA GCA GAA GAC GGC ATA CGA GAT TGC AAT GAG TGA CTG GAG TTC AGA CGT GTG CTC TTC CGA TC*T
59	CTGGCCAC	CAA GCA GAA GAC GGC ATA CGA GAT CTG GCC ACG TGA CTG GAG TTC AGA CGT GTG CTC TTC CGA TC*T
60	GATCCTGC	CAA GCA GAA GAC GGC ATA CGA GAT GAT CCT GCG TGA CTG GAG TTC AGA CGT GTG CTC TTC CGA TC*T
61	CACGCGTC	CAA GCA GAA GAC GGC ATA CGA GAT CAC GCG TCG TGA CTG GAG TTC AGA CGT GTG CTC TTC CGA TC*T
62	CATGGCAG	CAA GCA GAA GAC GGC ATA CGA GAT CAT GGC AGG TGA CTG GAG TTC AGA CGT GTG CTC TTC CGA TC*T
63	CTTCTCCG	CAA GCA GAA GAC GGC ATA CGA GAT CTT CTC CGG TGA CTG GAG TTC AGA CGT GTG CTC TTC CGA TC*T
64	GAAGGAAT	CAA GCA GAA GAC GGC ATA CGA GAT GAA GGA ATG TGA CTG GAG TTC AGA CGT GTG CTC TTC CGA TC*T
65	CAGCCAGA	CAA GCA GAA GAC GGC ATA CGA GAT CAG CCA GAG TGA CTG GAG TTC AGA CGT GTG CTC TTC CGA TC*T
66	GTGCATGA	CAA GCA GAA GAC GGC ATA CGA GAT GTG CAT GAG TGA CTG GAG TTC AGA CGT GTG CTC TTC CGA TC*T
67	TCTTGGAC	CAA GCA GAA GAC GGC ATA CGA GAT TCT TGG ACG TGA CTG GAG TTC AGA CGT GTG CTC TTC CGA TC*T
68	GTGTTATC	CAA GCA GAA GAC GGC ATA CGA GAT GTG TTA TCG TGA CTG GAG TTC AGA CGT GTG CTC TTC CGA TC*T
69	TGGTGTTC	CAA GCA GAA GAC GGC ATA CGA GAT TGG TGT TCG TGA CTG GAG TTC AGA CGT GTG CTC TTC CGA TC*T
70	TGATTGAG	CAA GCA GAA GAC GGC ATA CGA GAT TGA TTG AGG TGA CTG GAG TTC AGA CGT GTG CTC TTC CGA TC*T
71	CTGAAGCG	CAA GCA GAA GAC GGC ATA CGA GAT CTG AAG CGG TGA CTG GAG TTC AGA CGT GTG CTC TTC CGA TC*T
72	AGCTTCAT	CAA GCA GAA GAC GGC ATA CGA GAT AGC TTC ATG TGA CTG GAG TTC AGA CGT GTG CTC TTC CGA TC*T
73	ACTGCAGA	CAA GCA GAA GAC GGC ATA CGA GAT ACT GCA GAG TGA CTG GAG TTC AGA CGT GTG CTC TTC CGA TC*T
74	CATGATGA	CAA GCA GAA GAC GGC ATA CGA GAT CAT GAT GAG TGA CTG GAG TTC AGA CGT GTG CTC TTC CGA TC*T
75	CTCCGGAC	CAA GCA GAA GAC GGC ATA CGA GAT CTC CGG ACG TGA CTG GAG TTC AGA CGT GTG CTC TTC CGA TC*T
76	CATATATC	CAA GCA GAA GAC GGC ATA CGA GAT CAT ATA TCG TGA CTG GAG TTC AGA CGT GTG CTC TTC CGA TC*T
77	GTTAGTTC	CAA GCA GAA GAC GGC ATA CGA GAT GTT AGT TCG TGA CTG GAG TTC AGA CGT GTG CTC TTC CGA TC*T
78	GTCATGAG	CAA GCA GAA GAC GGC ATA CGA GAT GTC ATG AGG TGA CTG GAG TTC AGA CGT GTG CTC TTC CGA TC*T
79	TCAGAGCG	CAA GCA GAA GAC GGC ATA CGA GAT TCA GAG CGG TGA CTG GAG TTC AGA CGT GTG CTC TTC CGA TC*T
80	GATCTCAT	CAA GCA GAA GAC GGC ATA CGA GAT GAT CTC ATG TGA CTG GAG TTC AGA CGT GTG CTC TTC CGA TC*T
81	GTATGCGA	CAA GCA GAA GAC GGC ATA CGA GAT GTA TGC GAG TGA CTG GAG TTC AGA CGT GTG CTC TTC CGA TC*T
82	CTGAGATA	CAA GCA GAA GAC GGC ATA CGA GAT CTG AGA TAG TGA CTG GAG TTC AGA CGT GTG CTC TTC CGA TC*T
83	CTAATTAC	CAA GCA GAA GAC GGC ATA CGA GAT CTA ATT ACG TGA CTG GAG TTC AGA CGT GTG CTC TTC CGA TC*T
84	ACACTATC	CAA GCA GAA GAC GGC ATA CGA GAT ACA CTA TCG TGA CTG GAG TTC AGA CGT GTG CTC TTC CGA TC*T
85	CAACGTTC	CAA GCA GAA GAC GGC ATA CGA GAT CAA CGT TCG TGA CTG GAG TTC AGA CGT GTG CTC TTC CGA TC*T
86	CAGCTGAG	CAA GCA GAA GAC GGC ATA CGA GAT CAG CTG AGG TGA CTG GAG TTC AGA CGT GTG CTC TTC CGA TC*T
87	CTCTCTCG	CAA GCA GAA GAC GGC ATA CGA GAT CTC TCT CGG TGA CTG GAG TTC AGA CGT GTG CTC TTC CGA TC*T
88	GAGAAGAT	CAA GCA GAA GAC GGC ATA CGA GAT GAG AAG ATG TGA CTG GAG TTC AGA CGT GTG CTC TTC CGA TC*T
89	CACAGCGA	CAA GCA GAA GAC GGC ATA CGA GAT CAC AGC GAG TGA CTG GAG TTC AGA CGT GTG CTC TTC CGA TC*T
90	TCAGGATA	CAA GCA GAA GAC GGC ATA CGA GAT TCA GGA TAG TGA CTG GAG TTC AGA CGT GTG CTC TTC CGA TC*T
91	TCGGTTAC	CAA GCA GAA GAC GGC ATA CGA GAT TCG GTT ACG TGA CTG GAG TTC AGA CGT GTG CTC TTC CGA TC*T
92	TGCGTATC	CAA GCA GAA GAC GGC ATA CGA GAT TGC GTA TCG TGA CTG GAG TTC AGA CGT GTG CTC TTC CGA TC*T
93	ACCGGTTC	CAA GCA GAA GAC GGC ATA CGA GAT ACC GGT TCG TGA CTG GAG TTC AGA CGT GTG CTC TTC CGA TC*T
94	ACTGTGAG	CAA GCA GAA GAC GGC ATA CGA GAT ACT GTG AGG TGA CTG GAG TTC AGA CGT GTG CTC TTC CGA TC*T
95	TCTCCTCG	CAA GCA GAA GAC GGC ATA CGA GAT TCT CCT CGG TGA CTG GAG TTC AGA CGT GTG CTC TTC CGA TC*T
96	AGAGAGAT	CAA GCA GAA GAC GGC ATA CGA GAT AGA GAG ATG TGA CTG GAG TTC AGA CGT GTG CTC TTC CGA TC*T
Nextera Primer (Forward)		CAA GCA GAA GAC GGC ATA CGA GAT NNNNNNNN GTC TCG TGG GCT CGG
Nextera Dual Primer (Reverse)		AAT GAT ACG GCG ACC ACC GAG ATC TAC AC NNNNNNNN TCG TCG GCA GCG TC
